# Post-traumatic stress disorder and depression prevalence and associated risk factors among local disaster relief and reconstruction workers fourteen months after the Great East Japan Earthquake: a cross-sectional study

**DOI:** 10.1186/s12888-015-0440-y

**Published:** 2015-03-24

**Authors:** Atsushi Sakuma, Yoko Takahashi, Ikki Ueda, Hirotoshi Sato, Masahiro Katsura, Mikika Abe, Ayami Nagao, Yuriko Suzuki, Masako Kakizaki, Ichiro Tsuji, Hiroo Matsuoka, Kazunori Matsumoto

**Affiliations:** 1 Department of Psychiatry, Tohoku University Hospital, 1-1 Seiryo-machi, Aoba-ku, Sendai, Miyagi 980-8574 Japan; 2 Miyagi Disaster Mental Health Care Center, 2-18-21 Honcho, Aoba-ku, Sendai, Miyagi 980-0014 Japan; 3 Department of Preventive Psychiatry, Tohoku University Graduate School of Medicine, 2-1 Seiryo-machi, Aoba-ku, Sendai, Miyagi 980-8574 Japan; 4 Department of Psychiatry, Tohoku University Graduate School of Medicine, 2-1 Seiryo-machi, Aoba-ku, Sendai, Miyagi 980-8574 Japan; 5 Department of Adult Mental Health, National Institute of Mental Health, National Center of Neurology and Psychiatry, 4-1-1 Ogawa-Higashi, Kodaira, Tokyo 187-8551 Japan; 6 Division of Epidemiology, Department of Public Health and Forensic Medicine, Tohoku University Graduate School of Medicine, 2-1 Seiryo-machi, Aoba-ku, Sendai, Miyagi 980-8574 Japan

**Keywords:** Great East Japan Earthquake, Tsunami, Disaster relief worker, Firefighter, Local municipality worker, Medical worker, Post-traumatic stress disorder (PTSD), Depression, Psychological distress

## Abstract

**Background:**

Many local workers have been involved in rescue and reconstruction duties since the Great East Japan Earthquake (GEJE) on March 11, 2011. These workers continuously confront diverse stressors as both survivors and relief and reconstruction workers. However, little is known about the psychological sequelae among these workers. Thus, we assessed the prevalence of and personal/workplace risk factors for probable post-traumatic stress disorder (PTSD), probable depression, and high general psychological distress in this population.

**Methods:**

Participants (N = 1294; overall response rate, 82.9%) were workers (firefighters, n = 327; local municipality workers, n = 610; hospital medical workers, n = 357) in coastal areas of Miyagi prefecture. The study was cross-sectional and conducted 14 months after the GEJE using a self-administered questionnaire which included the PTSD Checklist–Specific Version, the Patient Health Questionnaire-9, and the K6 scale. Significant risk factors from bivariate analysis, such as displacement, dead or missing family member(s), near-death experience, disaster related work, lack of communication, and lack of rest were considered potential factors in probable PTSD, probable depression, and high general psychological distress, and were entered into the multivariable logistic regression model.

**Results:**

The prevalence of probable PTSD, probable depression, and high general psychological distress was higher among municipality (6.6%, 15.9%, and 14.9%, respectively) and medical (6.6%, 14.3%, and 14.5%, respectively) workers than among firefighters (1.6%, 3.8%, and 2.6%, respectively). Lack of rest was associated with increased risk of PTSD and depression in municipality and medical workers; lack of communication was linked to increased PTSD risk in medical workers and depression in municipality and medical workers; and involvement in disaster-related work was associated with increased PTSD and depression risk in municipality workers.

**Conclusions:**

The present results indicate that at 14 months after the GEJE, mental health consequences differed between occupations. High preparedness, early mental health interventions, and the return of ordinary working conditions might have contributed to the relative mental health resilience of the firefighters. Unlike the direct effects of disasters, workplace risk factors can be modified after disasters; thus, we should develop countermeasures to improve the working conditions of local disaster relief and reconstruction workers.

## Background

Survivors of natural or manmade disasters experience physical and psychological distress, and previous studies have shown that post-traumatic stress disorder (PTSD), depression, and other mental health problems may increase after such disasters [1,2]. Large-scale natural disasters in particular involve an extraordinarily large number of people and not only affect the mental health of direct victims and the general population, but also workers who perform rescue and recovery duties [3,4].

Many studies thus far have focused on the psychological sequelae of disaster rescue workers or “traditional” first responders [4], such as police officers, firefighters, emergency medical technicians, and military personnel, who are trained and have a responsibility to save citizens’ lives. Such studies have found that PTSD prevalence among first responders ranges from 10 to 20%, which is intermediate between the prevalence rates of direct victims of disasters (30 to 40%) and the general population (5 to 10%) [5,6]. A study of rescue/recovery workers who responded to the World Trade Center (WTC) disaster revealed that risk of mental health problems may differ among occupations, and that occupations least likely to have had prior disaster training were at greater risk [7]. Furthermore, physical and mental health problems have been shown to persist for at least 9 years in this population [8].

While the mental health of traditional first responders has been explored, fewer studies have examined this in other disaster relief workers, such as local government workers, medical service personnel, health care workers, teachers, and social workers, who are instrumental in the reconstruction of community functions in devastated areas [4,9]. Following large-scale natural disasters, it is often the case that these responders themselves are disaster survivors, and are thus suffering from personal trauma and losses. They may have experienced immediate life-threatening dangers, witnessed tragic events, experienced loss of family or friends, lost possessions, or been forced to relocate [9]. Nonetheless, immediately after the disaster, many continuously work to assist victims and restore the local community. In other words, these workers play a dual role: disaster survivors and support providers. In contrast to traditional first responders, who are temporarily deployed to affected areas, these local workers confront diverse stressors for a longer period of time; thus, it is more challenging for them to maintain a balance between work and daily life [9]. Therefore, the mental health status of these individuals may differ from that of traditional first responders or disaster survivors in the general population. Since such local workers play a vital role in the relief and reconstruction of disaster-affected areas, the maintenance of their mental health following a large-scale disaster is a critical issue. Yet, little is known about the prevalence of PTSD, depression, and high psychological distress and associated risk factors among local workers involved in relief and reconstruction activities for a long period of time as members of the affected community.

The Great East Japan Earthquake (GEJE) struck the Northeastern part of Japan on March 11, 2011. It was one of the largest earthquakes ever recorded in Japan, measuring 9.0 on the Richter scale. The earthquake triggered a huge tsunami, which killed more than 18,000 people and destroyed approximately 400,000 houses. In Miyagi prefecture, which was closest to the earthquake epicenter, more than 10,000 lives were lost. All the cities and towns in the coastal regions of the prefecture were severely damaged [10], and many local workers have been involved in relief and reconstruction of the affected areas since the disaster. Because such a large area was devastated by the GEJE, the process of local community reconstruction has been lengthily delayed, and the mental health of local public workers has become a great concern [11].

In this study, we investigated the mental health conditions of local workers in the coastal area of the Miyagi prefecture 14 months after the GEJE. We aimed to assess the prevalence of probable PTSD, probable depression, and high psychological distress among different occupations: firefighters, municipality workers, and hospital medical workers. We also examined whether personal disaster-related experience and work-related stressors were associated with an increased risk of PTSD, depression, or psychological distress.

## Method

In this cross-sectional study, mental health conditions and related factors were assessed among local municipality workers, medical workers, and firefighters in the tsunami-affected area of the GEJE.

The study was conducted in May 2012 (i.e., 14 months after the earthquake) using a self-administered questionnaire. Workers were eligible to participate if they were already employed in the coastal area of Miyagi prefecture at the time of the GEJE. Workers who began working there after the GEJE, or workers who were dispatched to Miyagi prefecture from other municipalities were excluded from the study. A total of 1294 workers who met the criteria and responded to a sufficient number of questions to accurately screen for probable PTSD, probable depression, and high general psychological distress were included in the study.

### Participants

The study sample consisted of local municipality workers of district A, medical workers of district A, and firefighters of district B. District A and B are adjacent coastal areas in Miyagi prefecture and share similar geographical features. These two areas were among the hardest hit by the tsunami. In both districts, about 2% of the population was lost, and more than 30% of the houses collapsed entirely. The damage in both districts was much higher than the average in Miyagi prefecture (0.5% of the population lost; 9% of houses totally collapsed). The sample size of each workplace was determined by the number of workers who kept working from the time of the GEJE to the time of the survey.

#### Local municipality workers (N = 610)

Local municipalities play a central role in disaster response activities and the reconstruction of disaster-affected areas. From immediately after the onset of the GEJE, municipality workers were involved in disaster response and restoration activities, including management of evacuation centers, damage assessment, management of temporary morgues, disposal of disaster debris, and restoration of public services. Subsequently, they engaged in long-term reconstruction activities, including building and managing temporary houses, restoring damaged homes, constructing infrastructure, reconstructing industry, and providing health services to victims.

#### Hospital medical workers (N = 421)

Hospital medical workers included doctors (n = 2), nurses (n = 243), pharmacists (n = 3), medical technologists (n = 62), midwives (n = 10), and ancillary medical personnel (n = 37) who worked at a disaster base hospital in district A. This hospital played a central role in accepting and transporting injured and sick people after the disaster, and functioned as a headquarters for medical relief teams from different parts of Japan. Disaster relief activities by the medical workers and the medical relief teams lasted for as long as 6 months, since it took a significant amount of time for local clinics and hospitals in the affected areas to become functional again [12].

#### Firefighters (N = 327)

Firefighters were professional firefighters who worked in district B. In the acute phase following the disaster, they were involved in firefighting, emergency services, rescue operations, and searching for bodies.

### Assessment

Self-administered questionnaires were used to assess demographic characteristics (age, sex), personal risk factors, and workplace risk factors (Table 1; coded dichotomously as “yes” or “no”).

**Table 1:** **Participant characteristics, and incidence of probable PTSD, probable depression, and high general psychological distress**

	Total (N = 1294)			Local municipality workers (n = 610)			Hospital medical workers (n = 357)			Firefighters (n = 327)			P
	N	%	(95 % CI)	n	%	(95 % CI)	n	%	(95 % CI)	n	%	(95 % CI)	
**Age**													
20–39	517			208			122			187			
40–59	769	42.8	(10.6)^a^	398	44.9	(9.7)^a^	235	43.4	(9.6)^a^	136	38.7	(11.9)^a^	<0.001
60+	8			4			0			4			
**Sex**													
Male	720	56.2	(53.5-59.0)	353	57.9	(53.9-61.8)	40	11.2	(7.9-14.5)	327	100.0		<0.001
Female	574	44.4	(41.7-47.1)	257	42.1	(38.2-46.1)	317	88.8	(85.5-92.1)	0	0.0		
**Workplace factors**													
Supervisory work status	142	11.0	(9.3-12.8)	87	14.3	(11.5-17.0)	28	7.8	(5.0-10.6)	27	8.3	(5.3-11.3)	<0.01
Mainly disaster-related work	312	24.5	(22.1-26.8)	196	32.5	(28.7-36.2)	16	4.6	(2.4-6.8)	100	30.7	(25.7-35.8)	<0.001
Lack of communication	252	17.2	(15.1-19.2)	136	20.4	(17.3-23.5)	72	18.9	(14.9-22.8)	44	7.5	(4.6-10.3)	<0.001
Lack of rest	516	39.0	(36.3-41.7)	272	40.8	(37.1-44.6)	181	47.6	(42.6-52.7)	63	19.3	(14.9-23.6)	<0.001
Dead or missing colleague(s)	412	32.2	(29.7-34.8)	109	17.9	(14.8-20.9)	45	12.6	(9.1-16.1)	258	79.8	(75.4-84.2)	<0.001
**Personal factors**													
Displacement	301	23.3	(20.9-25.6)	137	22.6	(19.3-25.9)	95	27.0	(22.3-31.6)	69	21.4	(16.9-25.9)	0.16
Dead or missing family member(s)	109	8.5	(7.0-10.0)	39	6.4	(4.4-8.3)	36	10.1	(6.9-13.2)	34	10.6	(7.2-13.9)	<0.05
Near-death experience	696	54.4	(51.7-57.2)	295	48.7	(44.7-52.7)	230	65.7	(60.7-70.7)	171	52.5	(47.0-58.0)	<0.001
**Probable PTSD** ^b^													
Diagnostic criteria	74	6.2	(4.8-7.5)	43	7.7	(5.5-9.9)	24	7.2	(4.4-10.0)	7	2.3	(0.6-3.9)	<0.01
Cutoff score	89	7.4	(5.9-8.9)	50	9.0	(6.6-11.3)	31	9.3	(6.2-12.4)	8	2.6	(0.8-4.4)	<0.01
Both of the above	64	5.3	(4.1-6.6)	37	6.6	(4.4-8.6)	22	6.6	(3.1-8.2)	5	1.6	(0.2-3.0)	<0.01
Adjusted odds ratio					4.0	(1.6-10.4)		3.8	(1.4-10.2)		1.0		
**Probable depression** ^c^													
Diagnostic criteria	188	15.2	(13.2-17.3)	112	19.1	(15.9-22.3)	61	18.2	(14.0-22.3)	15	4.7	(2.4-7.0)	<0.001
Cutoff score	235	19.3	(17.0-21.5)	143	24.4	(20.9-27.9)	74	22.0	(17.6-26.5)	18	5.6	(3.1-8.2)	<0.001
Both of the above	153	12.4	(10.5-14.3)	93	15.9	(13.2-19.4)	48	14.3	(10.3-18.1)	12	3.8	(1.7-5.9)	<0.001
Adjusted odds ratio					4.5	(2.4-8.4)		4.2	(2.2-7.9)		1.0		
**High general psychological distress** ^d^													
Cutoff score	143	11.4	(9.6-13.2)	86	14.9	(11.9-17.8)	49	14.5	(10.7-18.3)	8	2.6	(0.8-4.3)	<0.001
Adjusted odds ratio					6.2	(2.9-12.9)		5.9	(2.8-12.6)		1.0		

^a^Mean (SD) values are presented.

^b^Data were missing for 94 participants (7.4% of total; local municipality workers, n = 52; hospital medical workers, n = 24; firefighters; n = 18).

^c^Data were missing for 53 participants (4.1% of total; local municipality workers, n = 24; hospital medical workers, n = 21; firefighters; n = 8).

^d^Data were missing for 69 participants (5.3% of total; local municipality workers, n = 31; hospital medical workers, n = 19; firefighters; n = 19).

### Risk factors

Personal factors were as follows: 1) “displacement,” or whether the worker was displaced from prior housing to temporary or another type of housing (e.g., a relative’s house) because of the damage caused by the earthquake or tsunami; 2) “dead or missing family member(s),” or whether the worker’s family member was killed or still missing; and 3) “near-death experience,” or whether the worker had experienced a life-threatening situation due to the earthquake or tsunami.

Workplace factors were as follows: 1) “supervisory work status,” or whether the worker’s position was higher than that of the manager; 2) “mainly disaster-related work,” or whether the worker spent more than half of his or her occupational effort on disaster-related duties since the earthquake; 3) “dead or missing colleague(s),” or whether the worker’s colleagues were killed or still missing; 4) “lack of communication,” or whether the worker felt at the time of survey that workplace communication was lacking; and 5) “lack of rest,” or whether the worker felt at the time of survey that he or she was not obtaining sufficient rest because of occupational duties.

Symptoms of PTSD, depression, and general psychological distress were assessed using the PTSD Checklist–Specific Version (PCL-S) [13], the Patient Health Questionnaire-9 (PHQ-9) [14], and the K6 scale, respectively [15].

### Symptoms of probable PTSD

We used the Japanese version of the PCL-S, which was developed through a translation and back-translation process by one of authors (YS). The PCL-S consists of 17 items that correspond to DSM-IV PTSD symptom criteria B (re-experiencing), C (avoidance/numbing), and D (hyperarousal) [16]. Each item contains a Likert-type response format ranging from 1 (“not at all”) to 5 (“extremely”), and total score ranges from 17 to 85.

In addition to the sum PTSD severity score, each response of 3 (“moderately”) or higher is considered indicative of symptom presence; thus, the PCL-S offers a categorical algorithm based on DSM-IV criteria [16]. Each symptom was assessed as event-specific (“as a result of the Great East Japan Earthquake and Tsunami”) and current (“within the last 30 days”).

In order to allow for comparison across studies, we categorized an individual as having probable PTSD if he or she met one of the following three criteria, as reported previously by Perrin et al. [7]: (1) DSM-IV diagnostic criteria (i.e., the presence of at least one re-experiencing and intrusive symptom, three avoidance symptoms, and two hyperarousal symptoms [17]); (2) a standard cutoff total score of 44 [18]; and (3) a combination of both. The most conservative criterion (3) was used in assessing PTSD risk factors.

### Symptoms of probable depression

We used the Japanese version of the PHQ-9, a validated [19] measure that has been widely used in the general population and in primary care settings [20,21]. The PHQ-9 consists of 9 items that correspond to DSM-IV criteria for a major depressive episode [16] and measure symptom frequency over the preceding 2 weeks using a 4-point Likert-type scale, with item scores ranging from 0 (“not at all”) to 3 (“nearly every day”). The PHQ-9 total score ranges from 0 to 27.

In addition to the sum score of depression severity, each response of 2 (more than half days) or higher was considered indicative of symptom presence (except for suicidal thoughts, which was counted if present at all). Thus, the PHQ-9 offers a categorical algorithm based on DSM-IV criteria [16]: major depressive disorder or other depressive disorders are suspected when two or more of the indicated symptoms are present, with one of those symptoms being depressed mood or anhedonia.

We categorized a person as having probable depression if he or she met one of the following three criteria: (1) meeting DSM-IV criteria for major depressive disorder or another depressive disorder; (2) a total score of 10 [22] on the PHQ-9, which indicates moderately severe depression; and (3) a combination of both. The most conservative criterion (3) was used in assessing depression risk factors.

### General psychological distress

General psychological distress was evaluated using the K6 scale, Japanese version [23,24]. The K6 consists of 6 items that measure anxiety and depressive symptoms and uses a 5-point response format ranging from 0 (“none of the time”) to 4 (“all of the time”). Total score ranges from 0 to 24, with 13 or higher indicating high distress, and the potential presence of mood and anxiety disorders [24,25]. We identified those individuals with a total score of ≥13 as having a high level of general psychological distress.

In Japan, the K6 has been widely used to estimate psychological distress in the general population [26]. After the GEJE, the K6 was used to assess distress in disaster survivors [27,28], relief workers [29], and prefectural public servants [11,30]. Therefore, the use of the K6 enables us to compare psychological distress among different population groups.

### Statistical analysis

Descriptive analysis for demographic characteristics, prevalence rates for probable PTSD, probable depression, and high general psychological distress was conducted using SPSS version 20.0 (SPSS Inc., Chicago, Illinois). Two-tailed χ^2^ tests were used to evaluate differences in categorical variables, and one-way analysis of variance (ANOVA) was used to do so with continuous variables.

Firefighters were used as the referent category because previous studies have suggested that the prevalence of mental health problems among professional rescue workers is lower than that of non-professional rescuers [7,31] or direct victims of disaster [6].

Significant independent variables from bivariate analysis were considered potential factors in probable PTSD, probable depression, and high general psychological distress, and were entered into the multivariable logistic regression model (forced-entry method), as reported previously [29]. A two-sided P < 0.05 was used to indicate significance.

### Ethical issues

The data used in this study were acquired during health examinations conducted by each workplace. To protect the privacy of participants, the questionnaire was distributed and collected within each workplace by the person who oversaw the health of staff members. We obtained the electronic data, but not personal information. Therefore, we could not obtain written informed consent from each participant. Instead, we disclosed the study information, including the objectives and procedure, to the subjects and provided them with the opportunity to refuse participation. All participants who completed and returned the questionnaire were deemed to consent to the study. Moreover, the names of administrative regions, cities, or towns were anonymized so that the workplaces of the participants were not specified. The rights and welfare of participants were protected as per the ethical guidelines of the Declaration of Helsinki, and the ethical principles of the Ministry of Health, Labour, and Welfare of Japan were upheld. The study protocol and consent procedure was reviewed and approved by the Ethics Committee of Tohoku University Graduate School of Medicine (reference number: 2012-1-197).

## Results

### Demographic characteristics and prevalence of mental health problems

Table 1 shows the demographic characteristics, and the prevalence of probable PTSD, probable depression, and high general psychological distress.

### Demographic characteristics

Of the 1561 total workers, 1294 workers (82.9%) who returned the questionnaire were consequently included in the study (participant rates for municipality workers, hospital medical workers, and firefighters were 75.1%, 84.8%, and 99.7%, respectively). All participants had fully completed at least one of the three psychometric measures (i.e., PCL-S, PHQ-9, or K6).

High levels of personal damage were reported in these groups of workers. In total, 301 (23.3%) workers were displaced from their homes because of the damage caused by the GEJE. There were no significant differences in “displacement” among occupations. One hundred and nine (8.5%) workers lost family members, with firefighters having the highest percentage (10.6%) and municipality workers (6.4%) having the lowest among the worker groups. Six hundred and ninety-six (54.4%) participants reported “near-death experience”; medical workers (65.7%) were most likely and municipality workers (48.7%) were least likely to report such experiences.

Workplace factors differed significantly between occupations. A significantly greater percentage of municipality workers and medical workers reported “lack of communication” (20.4% and 18.9%, respectively) and “lack of rest” (40.8% and 47.6%, respectively), compared to firefighters (7.5%, 19.3%, respectively). Firefighters were most likely to report “dead or missing colleague(s)” (79.8%). “Mainly disaster-related work” was highest in municipality workers (32.5%), and lowest in medical workers (4.6%).

### Prevalence of probable PTSD, probable depression, and high general psychological distress

The prevalence of probable PTSD among municipality workers, medical workers, and firefighters with the most conservative combined criteria was 6.6% (adjusted odds ratio = 4.0), 6.6% (adjusted odds ratio = 4.0), and 1.6%, respectively. The prevalence among municipality workers and medical workers was significantly higher than that of firefighters.

The prevalence of probable depression among municipality workers, medical workers, and firefighters with the most conservative combined criteria was 15.9% (adjusted odds ratio = 4.5), 14.3% (adjusted odds ratio = 4.2), and 3.8%, respectively. The prevalence among municipality workers and medical workers was significantly higher than that of firefighters.

The prevalence of high general psychological distress among municipality workers, medical workers, and firefighters was 14.9% (adjusted odds ratio = 6.2), 14.5% (adjusted odds ratio = 5.9), and 2.6%, respectively. The prevalence among municipality workers and medical workers was significantly higher than that of firefighters.

### Risk factors for probable PTSD

Table 2 shows the results of bivariate and multivariate analysis of factors associated with probable PTSD. In firefighters, “lack of communication” (adjusted odds ratio = 8.98, P < 0.05) was the only factor associated with probable PTSD in bivariate analysis, and thus, multivariate analysis was not performed for this group. Multivariate analysis revealed that “lack of rest,” “dead or missing family member(s)”, and “near-death experience” was associated with probable PTSD in both municipality workers [adjusted odds ratio = 3.90 (P < 0.01), 4.37 (P < 0.01), and 2.72 (P < 0.05), respectively] and medical workers [adjusted odds ratio = 4.41 (P < 0.05), 5.29 (P < 0.01), and 6.38 (P < 0.05), respectively]. Probable PTSD was associated with “lack of communication” in medical workers (adjusted odds ratio = 3.70, P < 0.01), and “mainly disaster-related work” and “displacement” in municipality workers [adjusted odds ratio = 3.89 (P < 0.001) and 2.27 (P < 0.05), respectively]. “Age,” “female sex,” “supervisory work status,” and “dead or missing colleague(s)” was not associated with probable PTSD in any of the occupations in bivariate analysis.

**Table 2:** **Bivariate analysis and multivariate analysis of factors associated with probable PTSD**

	Local municipality workers				Hospital medical workers				Firefighters			
**Bivariate analysis**	β	SE	OR	P	β	SE	OR	P	β	SE	OR	P
Age	0.00	0.02	1.00	0.96	0.04	0.02	1.04	0.11	−0.02	0.04	0.98	0.66
Female sex	−0.26	0.37	0.77	0.48	0.63	0.75	1.87	0.41				
Workplace factors												
Supervisory work status	0.06	0.50	1.07	0.90	0.13	0.77	1.14	0.87	−17.18	8038.59	0.00	1.00
Mainly disaster-related work	1.54	0.38	4.68	<0.001	0.61	0.78	1.84	0.43	−0.57	1.13	0.57	0.61
Lack of communication	0.44	0.39	1.56	0.26	1.12	0.44	3.06	<0.05	2.20	0.94	8.98	<0.05
Lack of rest	1.71	0.43	5.55	<0.001	1.76	0.56	5.81	<0.01	18.77	2562.61	141708318.91	0.99
Dead or missing colleague(s)	−0.13	0.46	0.87	0.77	0.95	0.50	2.59	0.06	17.35	4874.11	34226161.92	1.00
Personal factors												
Displacement	1.16	0.36	3.19	<0.01	0.21	0.47	1.23	0.66	−0.11	1.13	0.90	0.92
Dead or missing family member(s)	1.52	0.47	4.56	<0.01	1.45	0.49	4.25	<0.01	0.75	1.13	2.12	0.51
Near-death experience	0.79	0.37	2.20	<0.05	1.47	0.63	4.33	<0.05	17.74	3361.11	50801095.58	1.00
**Multivariate analysis**	β	SE	OR	P	β	SE	OR	P	β	SE	OR	P
Age												
Female sex												
Workplace factors												
Supervisory work status												
Mainly disaster-related work	1.36	0.40	3.89	<0.001								
Lack of communication					1.31	0.49	3.70	<0.01				
Lack of rest	1.36	0.45	3.90	<0.01	1.48	0.58	4.41	<0.05				
Dead or missing colleague(s)												
Personal factors												
Displacement	0.82	0.39	2.27	<0.05								
Dead or missing family member(s)	1.48	0.54	4.37	<0.01	1.67	0.55	5.29	<0.01				
Near-death experience	1.00	0.41	2.72	<0.05	1.85	0.77	6.38	<0.05				

SE = standard error, OR = adjusted odds ratio.

### Risk factors for probable depression

Table 3 shows the results of bivariate and multivariate analysis of factors associated with probable depression. In firefighters, “lack of rest” (adjusted odds ratio = 6.25, P < 0.01) was the only factor associated with probable depression in the bivariate analysis; thus, multivariate analysis was not conducted for this group. Multivariate analysis revealed that “lack of communication” showed the highest odds ratio and “lack of rest” showed the second highest odds ratio in municipality workers [adjusted odds ratio = 3.02 (P < 0.001) and 2.70 (P < 0.001), respectively] and medical workers [adjusted odds ratio = 3.11 (P < 0.001) and 2.93 (P < 0.001), respectively]. Furthermore, “mainly disaster-related work” and “near-death experience” were significant factors for municipality workers (adjusted odds ratio = 1.94, P < 0.05) and medical workers (adjusted odds ratio = 2.22, P < 0.05), respectively.

**Table 3:** **Bivariate analysis and multivariate analysis of factors associated with probable depression**

	Local municipality workers				Hospital medical workers				Firefighters			
**Bivariate analysis**	β	SE	OR	P	β	SE	OR	P	β	SE	OR	P
Age	0.00	0.01	1.00	0.88	0.03	0.02	1.04	<0.05	0.01	0.02	1.01	0.68
Female sex	−0.27	0.24	0.77	0.27	0.56	0.49	1.75	0.26				
Workplace factors												
Supervisory work status	0.17	0.32	1.18	0.61	−0.4	0.63	0.67	0.53	−0.02	1.06	0.98	0.99
Mainly disaster-related work	0.87	0.24	2.38	<0.001	−1.18	1.04	0.31	0.26	−0.33	0.68	0.72	0.63
Lack of communication	1.31	0.25	3.71	<0.001	1.17	0.32	3.22	<0.001	0.95	0.81	2.59	0.24
Lack of rest	1.32	0.26	3.73	<0.001	1.22	0.32	3.39	<0.001	1.83	0.60	6.25	<0.01
Dead or missing colleague(s)	−0.29	0.32	0.75	0.37	−0.41	0.50	0.66	0.41	0.28	0.79	1.32	0.73
Personal factors												
Displacement	0.58	0.26	1.78	<0.05	0.24	0.32	1.27	0.46	1.01	0.60	2.76	0.09
Dead or missing family member(s)	0.96	0.38	2.62	<0.05	−0.35	0.55	0.70	0.52	0.54	0.80	1.72	0.50
Near-death experience	0.00	0.23	1.00	0.99	0.77	0.35	2.16	<0.05	0.22	0.60	1.25	0.71
**Multivariate analysis**	β	SE	OR	P	β	SE	OR	P	β	SE	OR	P
Age					0.03	0.02	1.03	0.05				
Female sex												
Workplace factors												
Supervisory work status												
Mainly disaster-related work	0.66	0.25	1.94	<0.05								
Lack of communication	1.11	0.26	3.02	<0.001	1.14	0.34	3.11	<0.001				
Lack of rest	0.99	0.27	2.70	<0.001	1.07	0.33	2.93	<0.001				
Dead or missing colleague(s)												
Personal factors												
Displacement	0.32	0.28	1.38	0.25								
Dead or missing family member(s)	0.76	0.44	2.14	0.08								
Near-death experience					0.80	0.37	2.22	<0.05				

SE = standard error, OR = adjusted odds ratio.

Although “displacement,” “dead or missing family member(s),” and “age” were significantly associated with probable depression in municipality workers in the bivariate analysis, none of these survived multivariate analysis. “Female sex,” “supervisory work status,” and “dead or missing colleague(s)” were not associated with probable depression in any of the occupations in the bivariate analysis.

### Risk factors for high general psychological distress

Table 4 shows the results of bivariate and multivariate analysis of factors associated with high general psychological distress. Multivariate analysis revealed that, among workplace factors, “lack of communication” was associated with psychological distress in medical workers and firefighters [adjusted odds ratio = 2.75 (P < 0.01) and 13.41 (P < 0.01), respectively]; “lack of rest” was a factor for municipality and medical workers [adjusted odds ratio = 3.90 (P < 0.001) and 2.31 (P < 0.05), respectively]; and “mainly disaster-related work” was a factor for municipality workers (adjusted odds ratio = 3.89, P < 0.01). Among personal factors, “dead or missing family member(s)” was associated with psychological distress in municipality workers and firefighters [adjusted odds ratio = 4.37 (P < 0.01) and 11.11 (P < 0.01), respectively]; “near-death experience” was a factor for municipality and medical workers [adjusted odds ratio = 2.72 (P < 0.05) and 2.53 (P < 0.05), respectively]; and “displacement” was a factor for municipality workers (adjusted odds ratio = 2.27, P < 0.05).

**Table 4:** **Bivariate analysis and multivariate analysis of factors associated with high general psychological distress**

	Local municipality workers				Hospital medical workers				Firefighters			
**Bivariate analysis**	β	SE	OR	P	β	SE	OR	P	β	SE	OR	P
Age	0.00	0.02	1.00	0.96	0.02	0.02	1.02	0.14	0.01	0.03	1.01	0.63
Female sex	−0.26	0.37	0.77	0.48	1.59	0.74	4.90	<0.05				
Workplace factors												
Supervisory work status	0.07	0.50	1.07	0.90	−0.05	0.56	0.95	0.93	−17.67	8038.59	0.00	1.00
Mainly disaster-related work	1.54	0.38	4.68	<0.001	0.09	0.64	1.09	0.90	−1.18	1.08	0.31	0.27
Lack of communication	0.44	0.39	1.56	0.26	0.97	0.32	2.65	<0.01	2.75	0.75	15.67	<0.001
Lack of rest	1.71	0.43	5.55	<0.001	1.05	0.32	2.87	<0.01	1.49	0.72	4.44	<0.05
Dead or missing colleague(s)	−0.13	0.46	0.88	0.77	−0.66	0.54	0.52	0.22	0.64	1.08	1.90	0.55
Personal factors												
Displacement	1.16	0.36	3.19	<0.01	0.40	0.32	1.49	0.21	−0.63	1.08	0.54	0.56
Dead or missing family member(s)	1.52	0.47	4.56	<0.01	0.75	0.41	2.12	0.07	2.20	0.73	9.00	<0.01
Near-death experience	0.79	0.37	2.20	<0.05	1.02	0.37	2.77	<0.01	1.02	0.83	2.77	0.22
**Multivariate analysis**	β	SE	OR	P	β	SE	OR	P	β	SE	OR	P
Age												
Female sex					1.34	0.76	3.81	0.08				
Workplace factors												
Supervisory work status												
Mainly disaster-related work	1.36	0.40	3.89	<0.01								
Lack of communication					1.01	0.34	2.75	<0.01	2.60	0.89	13.41	<0.01
Lack of rest	1.36	0.45	3.90	<0.001	0.84	0.33	2.31	<0.05	0.92	0.86	2.51	0.28
Dead or missing colleague(s)												
Personal factors												
Displacement	0.82	0.39	2.27	<0.05								
Dead or missing family member(s)	1.48	0.54	4.37	<0.01					2.41	0.83	11.11	<0.01
Near-death experience	1.00	0.41	2.72	<0.05	0.93	0.38	2.53	<0.05				

SE = standard error, OR = adjusted odds ratio.

Although “female sex” and “lack of rest” were significantly associated with psychological distress in medical workers and firefighters, respectively, in the bivariate analysis, these factors did not survive multivariate analysis. “Age,” “supervisory work status,” and “dead or missing colleague(s)” were not associated with high general psychological distress in any of the occupations in bivariate analysis.

## Discussion

To the best of our knowledge, this is the first study to investigate the prevalence of and risk factors for probable PTSD, depression, and high general psychological distress in local workers engaged in lengthy relief and reconstruction projects following a large-scale natural disaster. These workers were living in the disaster-affected community as survivors and serving as disaster relief and reconstruction workers at the same time. As community reconstruction can take years, it is crucial to ensure that the mental health of these local workers is maintained.

The present results show that 14 months after the GEJE, the consequences of the disaster on workers’ mental health differed across occupations: the prevalence of probable PTSD, depression, and high general psychological distress was significantly greater among municipality workers and medical workers compared to firefighters. Furthermore, the prevalence of high general psychological distress among municipality workers and medical workers was higher than that of survivors living in temporary housing or the general population living in tsunami-affected areas [27,28]. Workplace risk factors such as lack of rest, lack of communication, and involvement in disaster-related work affected risk of PTSD, depression, and high psychological distress differently in each occupation.

### Risk of PTSD

In the present study, the prevalence of probable PTSD in municipality workers and medical workers was 6.6%, which is much higher than the 12-month prevalence of PTSD in the general population in Japan (0.4%) [32]. In firefighters, PTSD prevalence was 1.6%, which is higher than that of the general population, but much lower than that of municipality and medical workers. The incidence of probable PTSD in the present sample was lower than that of firefighters, medical personnel, and government agencies 2 to 3 years after working at the WTC disaster site (12.2%, 11.6%, and 11.8%, respectively) [7]. The prevalence of PTSD is affected by type of disaster, and PTSD risk is reportedly lower after natural disasters than after human-made/technological disasters such as terror attacks [5,6]. Additionally, coastal areas of Miyagi prefecture have been repeatedly hit by huge tsunamis at intervals of several decades (i.e., at 1896, 1933, and 1960) [33], and people were culturally prepared (people of the area sustained effort to instill a culture of resilience and prevention based on continuous learning) to cope with tsunami disasters [34]. Thus, in these areas, past experience with disasters may have served as a moderator and consequently lessened the impact of the GEJE [35].

The lower PTSD risk among firefighters relative to municipality workers and medical workers observed in the present study is consistent with the finding that firefighters dispatched to the tsunami-affected area immediately after the GEJE did not exhibit PTSD symptoms [36]. Although we did not have any quantitative data on prior disaster training or experience for any of the studied occupations, we speculate that the lower risk of PTSD among firefighters may be partially explained by their prior training and experience with disaster [7,31].

After the Kobe earthquake in 1995, the importance of critical incident stress management programs for firefighters has been widely acknowledged and practiced in Japan [37,38], and such a program was provided to the present firefighters at their workplace on several occasions following the GEJE [38]. Thus, there is a possibility that these pre and post measures might have mitigated PTSD risk in this population. On the other hand, as is common to local municipal offices or hospitals in Japan, the workplace mental health care system was insufficient, and few workplaces had mental health support programs ready for implementation following a disaster.

In the present study, lack of communication was associated with increased PTSD risk in medical workers. To our knowledge, this is the first study that has demonstrated such a relationship in workers following a large-scale disaster. However, the importance of social support and sustained attachment to one’s social group during recovery from traumatic experiences has been repeatedly shown in previous studies [39-41]. Wang et al. [9] noted that feeling connected and positive at the workplace might be important for recovery from mass trauma and post-traumatic growth in local relief workers. Therefore, measures to promote communication at the workplace might facilitate psychological recovery in local medical workers.

At the time of survey, municipality and medical workers were more likely than firefighters to indicate lack of rest, and increased PTSD risk was associated with lack of rest among municipality and medical workers. Since the GEJE, municipality workers had been involved in large-scale and multi-year post-disaster reconstruction activities in addition to their ordinary duties, and chronic staff shortages have plagued most of the municipality offices in these areas [42]. Moreover, the exhaustion of medical workers has also been a concern. Since most local hospitals and clinics in the affected areas were damaged by the disaster, and a substantial number of them were permanently closed or still non-functional at the time of assessment, the number of emergency patients admitted to disaster base hospitals was increasing even 12 months after the GEJE [43]. Therefore, at the time of survey, municipality and medical workers were still suffering from increased workload and staff shortages; conversely, working conditions had normalized for firefighters by this time.

Factors indicating exposure to traumatic events as disaster survivors (i.e., dead or missing family member(s), displacement, or near-death experience) were associated with probable PTSD. This finding is consistent with those of previous studies showing that more severe exposure to a traumatic event is associated with more pronounced PTSD symptoms [5,44,45]. For example, losing family members or one’s home and possessions [29,46,47], or experiencing fear during a disaster might result in augmented PTSD symptoms [48].

### Risk of depression

In our participants, one in seven municipality and medical workers showed probable depression, which is approximately four to five times higher than the 12-month prevalence of major depression (3%) in the general population of Japan [32], indicating that depression risk may increase in some occupations among local workers. Although risk of depression among disaster-related workers has been studied less than that of PTSD, previous studies showed an increased risk of depression among disaster workers responding to an airplane crash [49] and the 9/11 WTC attacks [8].

On the other hand, depression risk was much lower in firefighters relative to municipality and medical workers, which is consistent with a previous finding [8] that depression risk differed between New York City police officers and other rescue and recovery workers one year after the 9/11 WTC attacks: the cumulative incidence of depression was 1.7% in the former and 10.8% in the latter. Probable depression in our sample was more prevalent than probable PTSD, which contradicts the findings of the abovementioned study [8] in which risk of PTSD was higher than that of depression. As mentioned above, we speculate that the lower risk of PTSD after natural disasters relative to human-made/technological disasters [5,6], and the historical and cultural experience with tsunami in these areas may explain the lower risk of PTSD relative to depression in our sample.

Workplace factors were strongly associated with probable depression. Depression was associated with lack of communication in municipality and medical workers, with involvement in disaster-related work in municipality workers, and with lack of rest in all three occupations. Under ordinary working conditions, depression is associated with long working hours [50] and poor interpersonal relationships at the workplace [51]; however, after a devastating disaster such as the GEJE, local workers have an enormous amount of work to do for a longer period of time, which reduces the time available for rest and interpersonal communication. While other disaster-related factors may additionally contribute to this reduction, the importance of rest [11] and a good relationship with coworkers [9,11] for local disaster-related workers has been previously demonstrated, and the present findings corroborate this notion in terms of preventing depression.

### Risk of general psychological distress

The prevalence of high general psychological distress among municipality (14.9%) and medical (14.5%) workers was more than twice that of the general population in Miyagi prefecture before the GEJE in 2010 (5.5%), and also higher than that of the general population of tsunami-devastated areas at 4 months (7.3%) [27], or displaced survivors living in temporary housing in Miyagi prefecture at 11 months after the GEJE (8.1%) [28]. On the other hand, the prevalence of high general psychological distress among firefighters was less than one-fifth that of municipality and medical workers, and less than that of the general population in the affected areas. This finding corresponds to the lower risk of PTSD and depression among firefighters in the present sample.

Similar to the present study, general psychological distress in public servants was examined in the Miyagi prefectural government at 7 months after the GEJE. The percentage of prefectural government workers who scored 13 or more on the K6 scale was 4.4% [11], which is slightly higher than that of local public servants (2.5%) [52], but much lower than that of the municipality workers in our study. Thus, although the prefectural and municipality government workers were working as public servants in the same disaster-affected prefecture and the former were investigated at a time closer to the disaster, severe psychological distress was more common in municipality workers. This is likely because, under the Japanese local government system, municipal government workers are required to be more directly involved in disaster-related work [11], and most of the present workers lived in the more severely damaged areas and had consequently experienced more direct loss and damage due to the GEJE.

### Limitations

There are several limitations to this study. First, since this study was cross-sectional and correlational, we cannot draw conclusions regarding the causation of the risk factors. Thus, our findings should be examined in future studies using prospective designs. Second, although several pre-disaster baseline risk factors, such as prior psychiatric problems, disaster experience, exposure to traumatic events, stress exposure, and alcohol consumption, are known to affect the mental health of affected people after a disaster [6,53], we could not obtain such data because they were beyond the scope of the workplace health examinations. As a result, we could not eliminate the possibility that the firefighters had had fewer mental health problems before the disaster. Third, because we used self-administered questionnaires to assess psychological symptoms and did not conduct a psychiatric diagnostic interview to confirm the results of the self-administered questionnaires, the prevalence of PTSD and depression could have been overestimated [54]. However, the present diagnostic estimation of PTSD and depression correspond to that reported in previous studies [2,7,55], enabling us to compare results from different studies. Fourth, we did not directly assess the degree of previous training experience and preparedness for disaster, participation in mental health interventions, or workload in each occupation; thus, we could not determine whether such factors might have been responsible for the differences in mental health conditions among the different occupations. Finally, the study questionnaires were distributed through the participants’ workplaces. Although we notified the participants that the results would remain confidential and would not be considered in performance evaluations, it is nonetheless possible that some participants may not have answered honestly because of the stigma attached to poor mental health [56].

## Conclusions

In this study, we examined mental health in local workers who were residents of the affected area and continuously involved in relief and reconstruction activities 14 months after a large-scale natural disaster, the GEJE. We found differences in PTSD and depression risk among the three local occupations: the risk was greater for municipality and medical workers than for firefighters. Although all workers were impacted by the disaster as members of the affected community and as local disaster relief and reconstruction workers, the effects of these circumstances may have been reduced in firefighters because of high preparedness, early mental health interventions, and a more prompt return of ordinary working conditions.

We revealed that work-related factors were strongly associated with increased risk of PTSD and depression. Lack of rest was associated with increased risk of PTSD and depression in municipality and medical workers; lack of communication was linked to increased risk of PTSD in medical workers and depression in municipality and medical workers; and involvement in disaster-related work was associated with increased risk of PTSD and depression in municipality workers. Unlike the direct effects of disasters, risk factors at the workplace, such as lack of communication and rest, can be modified after a disaster. Thus, we should develop countermeasures to improve working conditions for local disaster relief and reconstruction workers (e.g., developing educational programs or leaflets to inform workers of the psychological response after a disaster and stress management techniques, as well as to educate supervisory employees about the importance of staff rotation to prevent burn-out). Such interventions should be particularly geared towards promoting workplace communication and rest after a massive disaster.

## Abbreviations

PTSD: Post-traumatic stress disorder
GEJE: Great east Japan earthquake
PCL-S: PTSD Checklist–specific version
PHQ-9: Patient health questionnaire-9
